# Supplementary material for: Giant intrinsic photovoltaic effect in one-dimensional van der Waals grain boundaries
Source: Nat Commun. 2024 Jan 13;15:501. doi: 10.1038/s41467-024-44792-4 (PMC10787835; doi:10.1038/s41467-024-44792-4)
Supplement: Supplementary file 1 — Supplementary Information [file 41467_2024_44792_MOESM1_ESM.pdf]

Supplementary Information for "Giant intrinsic photovoltaic effect in one-dimensional van der Waals grain boundaries"

Yongheng Zhou[1,†], Xin Zhou[2,5†], Xiang-Long Yu[3,4,*], Zihan Liang[1], Xiaoxu Zhao[5], Taihong Wang[1], Jinshui Miao[6,*], Xiaolong Chen[1,*]

[1]Department of Electrical and Electronic Engineering, Southern University of Science and Technology, 1088 Xueyuan Avenue, Shenzhen 518055, China

[2]Department of Materials Science and Engineering, National University of Singapore, Singapore 117575, Singapore

[3]Shenzhen Institute for Quantum Science and Engineering, Southern University of Science and Technology, 1088 Xueyuan Avenue, Shenzhen 518055, China.

[4]International Quantum Academy, Shenzhen 518048, China.

[5]School of Materials Science and Engineering, Peking University, Beijing 100871, China

[6]State Key Laboratory of Infrared Physics, Shanghai Institute of Technical Physics, Chinese Academy of Sciences, Shanghai 200083, China

[†]These authors contributed equally

*Corresponding authors: Xiaolong Chen (chenxl@sustech.edu.cn); Xiang-Long Yu (yuxl@sustech.edu.cn); Jinshui Miao (jsmiao@mail.sitp.ac.cn)

Supplementary Figures

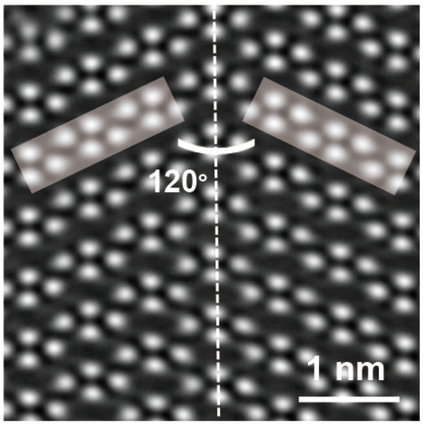

Supplementary Figure 1. The enlarged STEM image (after iFFT filter processed) of a ReS_2 GB, indicated by a white dashed line. The angle between Re-chain directions (denoted by white boxes) of two adjacent subdomains are ~120°.

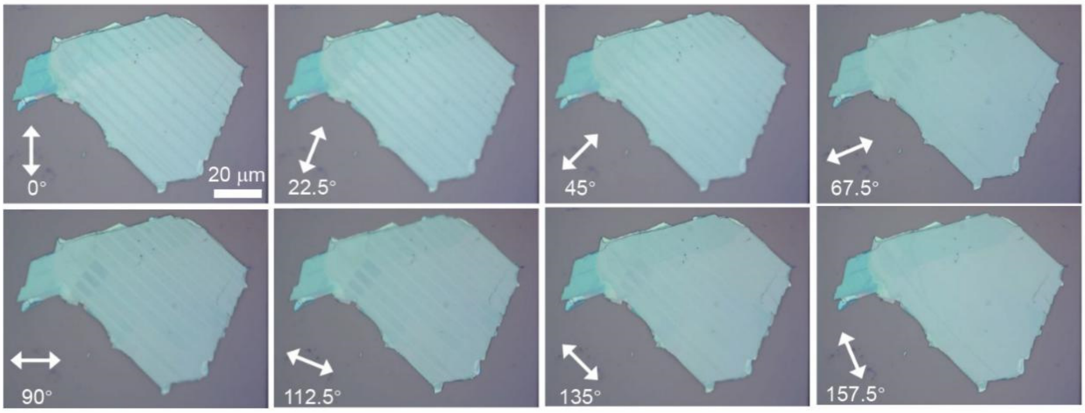

Supplementary Figure 2. Polarization-resolved optical microscopy images of a ReS_2 flake with GBs under different polarization angles from 0º to 157.5º.

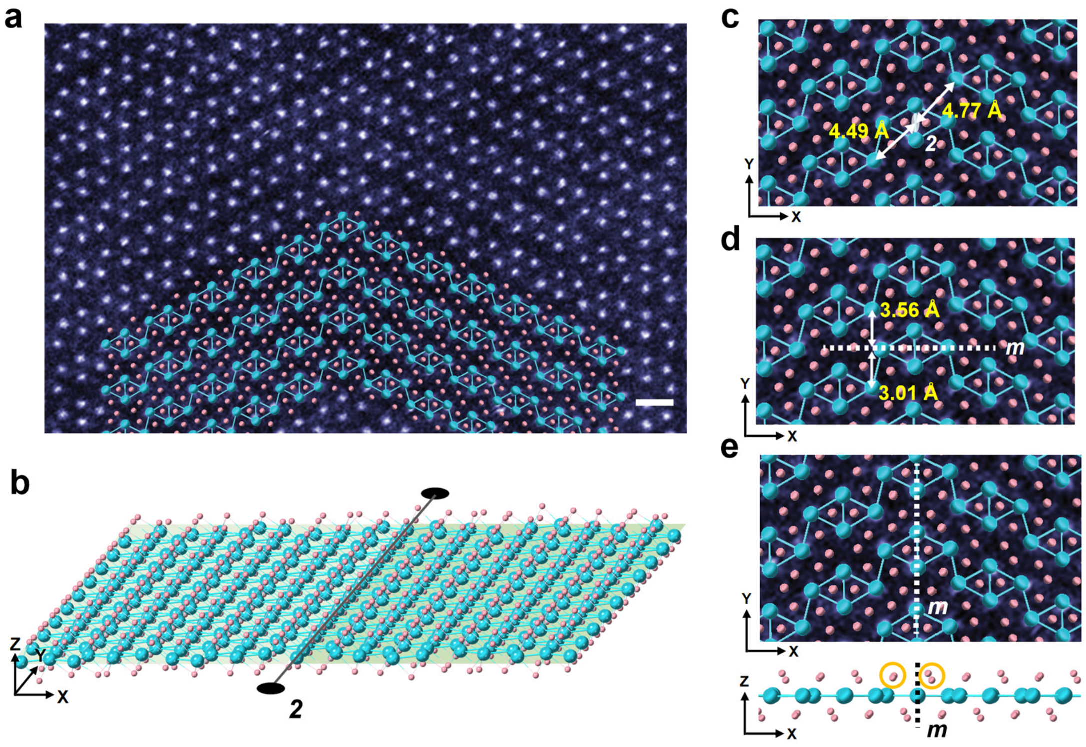

Supplementary Figure 3. **Symmetry analysis at ReS_2 GB**. **a**, Atomic-resolution STEM image of the boundary overlaid with the atomic model, where blue dots represent Re atoms and pink dots indicate S atoms. Scale bar: 5 Å. **b**, Detailed atomic model of the grain boundary highlighting the two-fold rotational axis along with the *y*-direction. **c**-**e**, Examination of other potential symmetry elements at the boundary: a possible two-fold rotational axis around the *z*-direction (c), a potential mirror plane parallel to the *xz* plane (d), and a possible mirror plane parallel to the *yz* plane (e). All these symmetry does not exist.

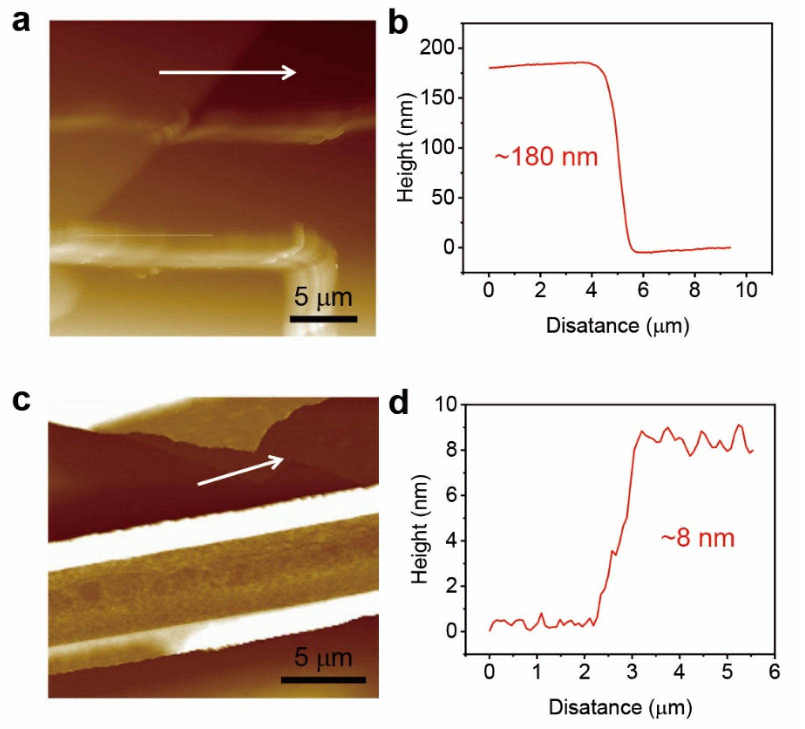

Supplementary Figure 4. **a**,**b**, Atomic force microscope (AFM) image of the 180 nm-thick ReS_2 flake. **c**,**d**, AFM image of the 8 nm-thick ReS_2 sample. The thickness of ReS_2 samples were taken along white solid lines in **a** and **c**. Scale bar is 5 μm.

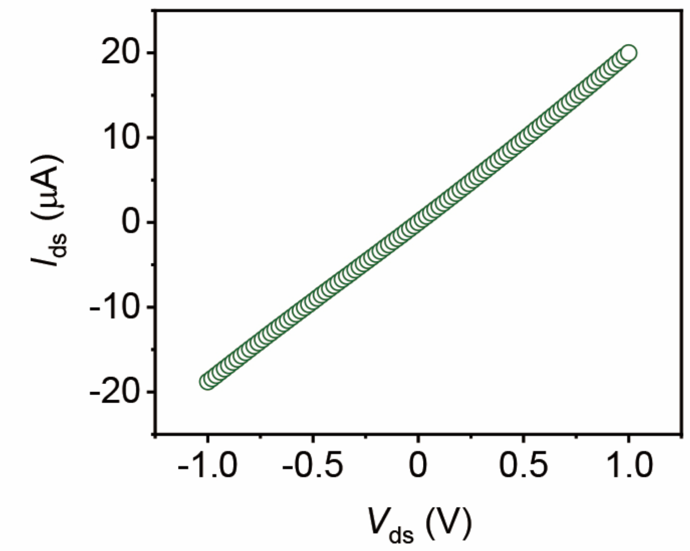

Supplementary Figure 5. **Characterizations of the 180 nm-thick ReS_2 flake device**. The device shows linear current-voltage characteristic, indicating a good Ohmic contact between ReS_2 and metal electrodes. The measurement was performed in vacuum and under dark condition.

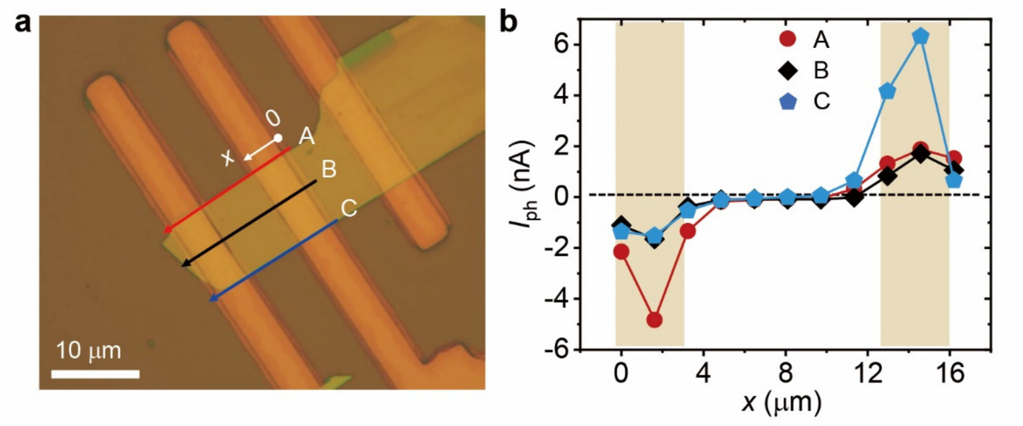

Supplementary Figure 6. **Characterizations of a ReS_2 sample without GBs. a**, Optical image of ReS_2 photodetector without GBs. Scale bar is 10 μm. **b**, I_{ph} measured along *x*-direction as indicated by the solid lines in **a**. Electrodes are marked by yellow regions. Vanishing photocurrent values are observed in the middle of ReS_2 channel.

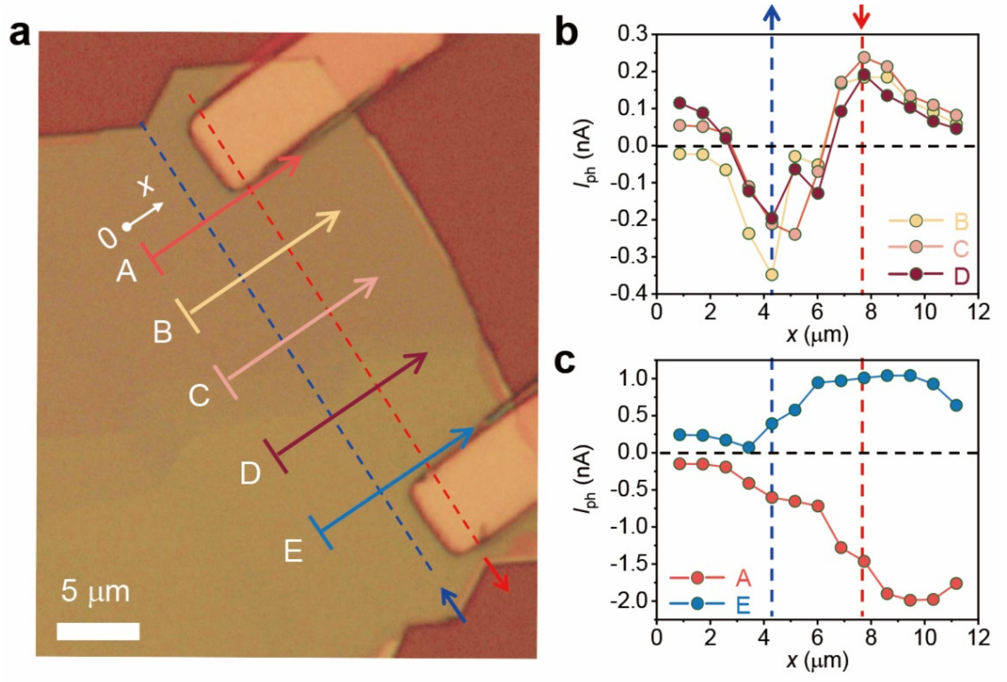

Supplementary Figure 7. **IPVE in another ReS_2 sample with GBs**. **a**, Optical image of ReS_2 photodetector. Two adjacent GBs with reversed orientations are denoted by "↑" and "↓" and marked by blue and red dash lines, respectively. Scale bar is 5 μm. **b**,**c**, Short-circuit photocurrents (I_{ph}) measured along x-direction as indicated by the solid lines in **a**. The peak/valley features are observed at two adjacent GBs along B, C and D lines, while are indistinguishable from strong extrinsic effects along A and E lines.

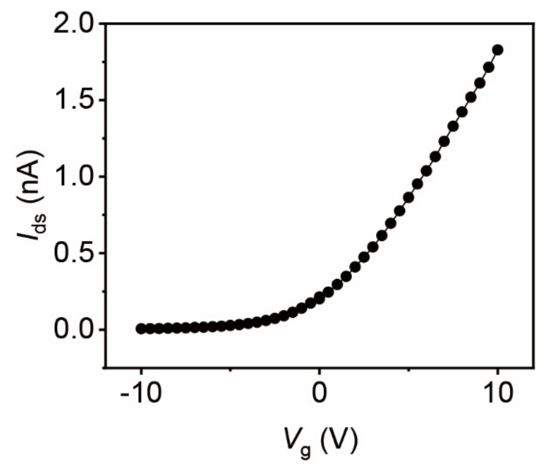

Supplementary Figure 8. Transfer curve of the 8 nm ReS_2 transistor. The source-drain voltage was fixed at 1 mV. The measurement was performed in vacuum and under dark condition.

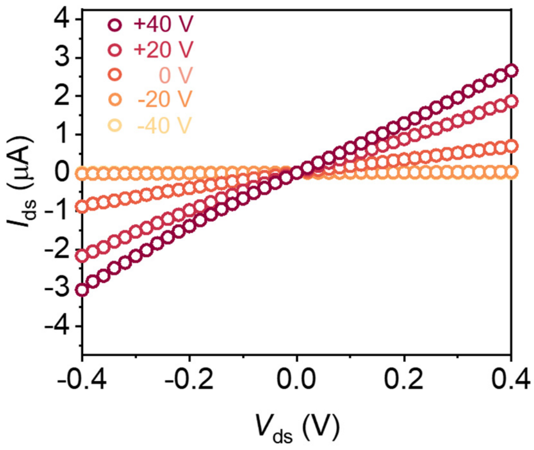

Supplementary Figure 9. I_{ds}–V_{ds} curves of the 8 nm ReS_2 device shows linear characteristic with gate voltage from -40 V to 40V.

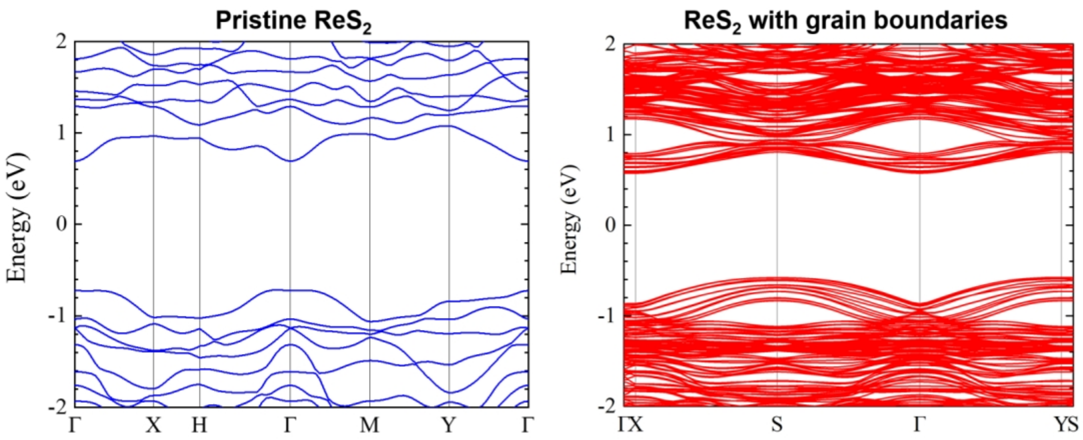

Supplementary Figure 10. Comparison of calculated band structures of pristine ReS_2 and ReS_2 with GBs. The unit cell of ReS_2 with GBs for calculation is shown in Supplementary Figure 14a below.

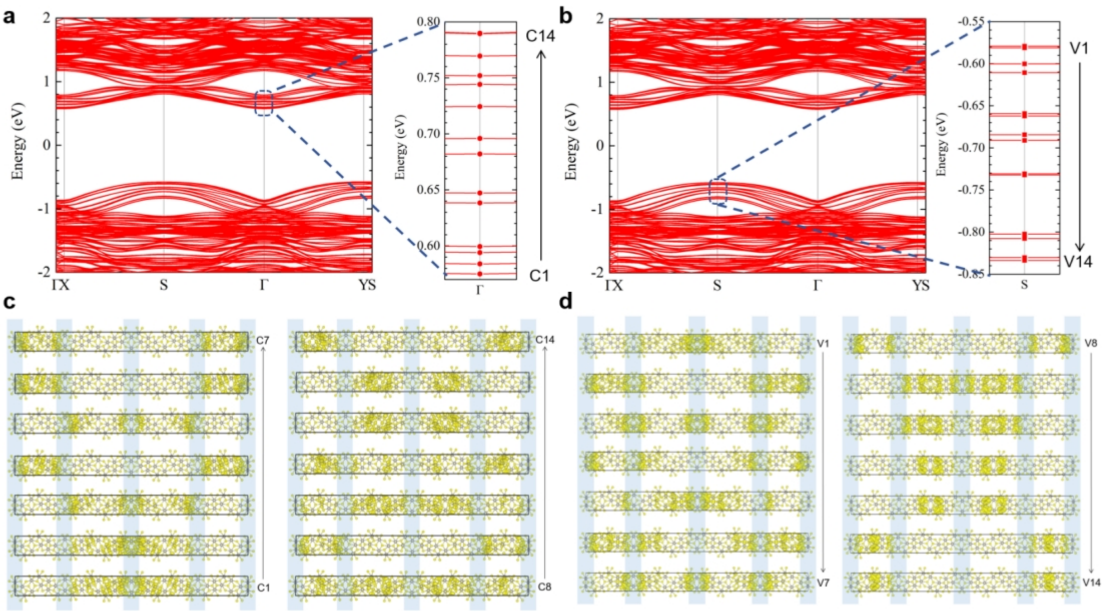

Supplementary Figure 11. The band properties and distribution of electron states of ReS_2 near GBs. a, **b**, Band structures of ReS_2 near GBs. 14 conduction bands (**a**) and 14 valence bands (**b**) near Fermi level were analyzed. **c**, **d**, Distribution of electron states contributed by conduction bands (**a**) and valence bands (**b**) near Fermi level. The GBs are marked by blue regions. Large number of electron states are found around GBs. The unit cell of ReS_2 with GBs for calculation is shown in Supplementary Figure 14a below.

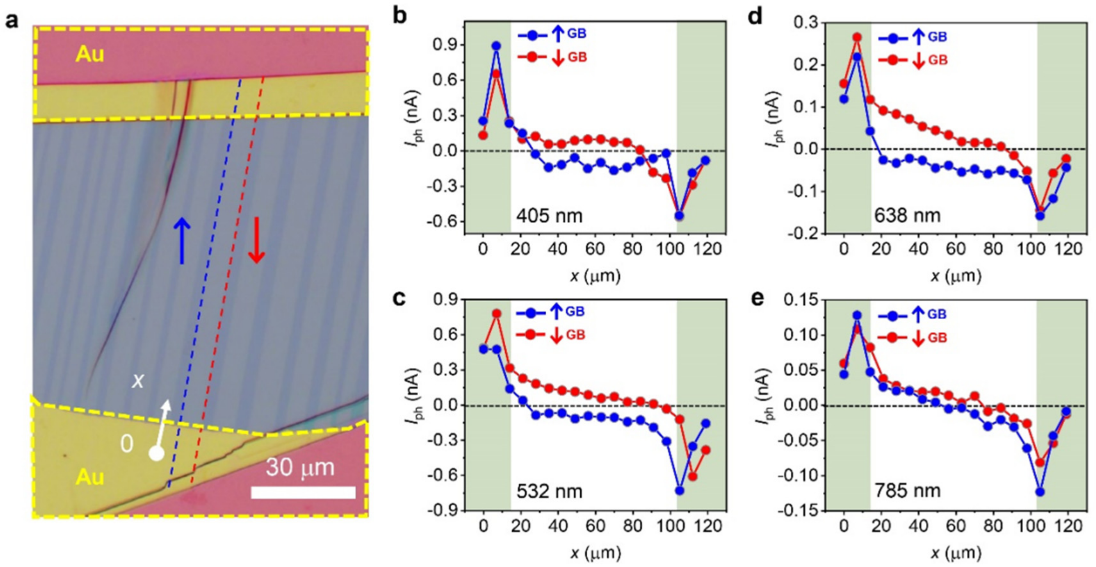

Supplementary Figure 12. **IPVE photocurrent at ReS_2 GBs with channel length over 100 μm**. **a**, The polarized optical image of the sample with grain boundary. The ↑ and ↓ GBs are marked by blue and red dash line, respectively. **b-e**, I_{ph} measured with incident 405, 532, 638 and 785 nm lasers along ↑ and ↓ GBs. Electrodes are marked by green regions. The power of all lasers was 200 μW.

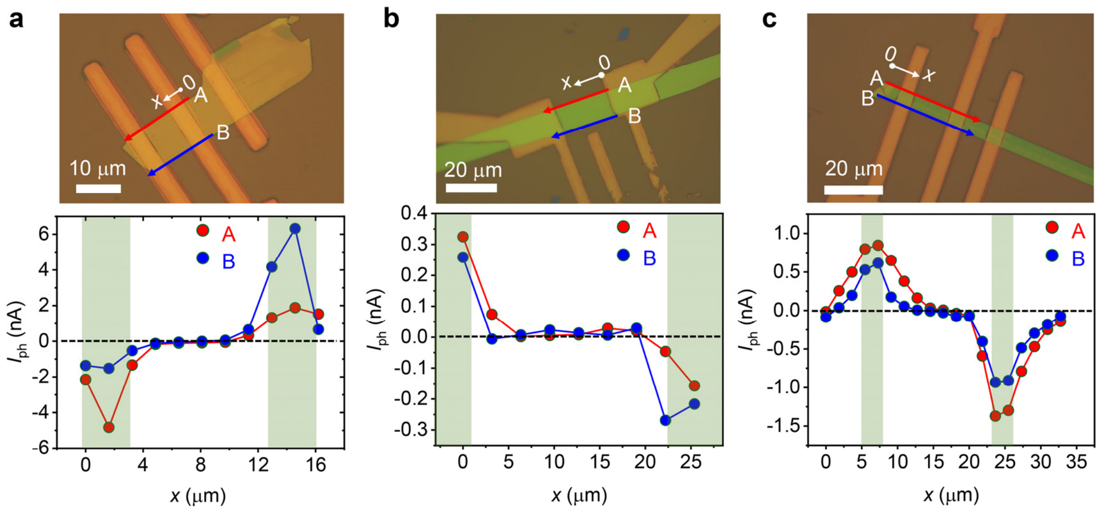

Supplementary Figure 13. Characterizations of edge photocurrents in ReS_2 samples without GBs. I_{ph} measured along the edges as indicated by the solid lines. Electrodes are marked by green shadows. The photocurrent vanished in the middle of ReS_2 channels.

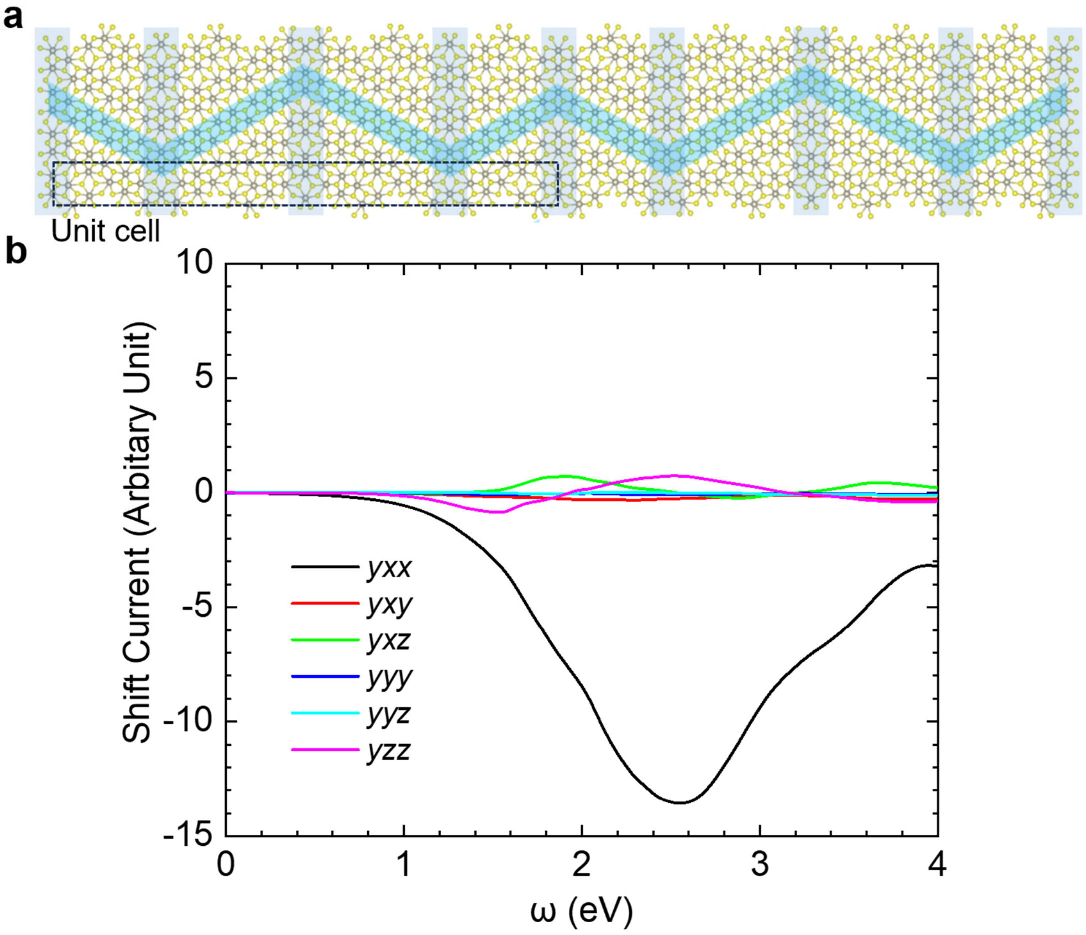

Supplementary Figure 14. **a**, Lattice structure of the simulated ReS_2 with GBs. The black box represents the unit cell. Dark blue and light blue mark Re chains and GBs, respectively. **b**, Calculated shift current along *y*-direction based on the construction of maximally localized Wannier functions using the WANNIER90 package.

Supplementary Note 1

The symmetry structure analysis of ReS_2 grain boundaries

The atomic configuration at the grain boundary (GB) with high-resolution STEM was examined and compared with the DFT-optimized atomic model (see Supplementary Fig. 3a). As shown in Supplementary Fig. 3b, the only symmetry present at GB is the two-fold rotation axis along the *y*-direction. Other possible symmetry elements at GBs were also evaluated. Supplementary Fig. 3c illustrates a potential two-fold rotational axis along the *z*-direction, which is found to be absent due to the non-equivalent distances from this axis to neighboring Re atoms (4.49 Å *vs*. 4.77 Å as indicated in Supplementary Fig. 3c). Additionally, the angle formed by Re atoms around this axis is 176.8°, not fulfilling the criteria for a C_2 rotational axis. Our examination of a mirror plane along the *xz* plane, as presented in Supplementary Fig. 3d, shows unequal distances from two neighboring Re atoms to this plane (3.56 Å *vs*. 3.01 Å). Finally, a potential mirror plane along the *yz* plane was considered. While it appears to exist from a top view, side view analysis clearly shows that such a mirror plane does not exist, especially in the arrangement of S atoms. In summary, the analysis results confirm that GB possesses only a two-fold rotational axis along *y*-direction, and the point group for the local structure around the boundary should be classified as C_2.

Supplementary Note 2

Theoretical investigation of bulk photovoltaic effects in ReS_2 grain boundaries

As discussed in the main test, under linearly polarized light, the DC photocurrent density $J_{\mathrm{l}}^{\mathrm{LBPVE}}$ propagates along *l*-direction (*l* represents *x*-, *y*-, or *z*-direction) can be written as[1, 2]

$$J_{\mathrm{l}}^{\mathrm{LBPVE}} = \frac{1}{2}\sum_{j,k} \chi_{\mathrm{ljk}} (E_{\mathrm{j}} E_{\mathrm{k}}^{*} + E_{\mathrm{k}} E_{\mathrm{j}}^{*}) \tag{1}$$

For light at normal-incidence (along *z*-direction), we have $E_z = 0$. Then we have

$$\overline{J^{\mathrm{LBPVE}}} = \begin{pmatrix} \chi_{\mathrm{xxx}} |E_x|^2 + \chi_{\mathrm{xyy}} |E_y|^2 + \chi_{\mathrm{xxy}} E_{\mathrm{x}} E_{\mathrm{y}}^{*} + \chi_{\mathrm{xxy}} E_y E_x^{*} \\ \chi_{\mathrm{yxx}} |E_x|^2 + \chi_{\mathrm{yyy}} |E_y|^2 + \chi_{\mathrm{yxy}} E_{\mathrm{x}} E_{\mathrm{y}}^{*} + \chi_{\mathrm{yxy}} E_y E_x^{*} \\ \chi_{\mathrm{zxx}} |E_x|^2 + \chi_{\mathrm{zyy}} |E_y|^2 + \chi_{\mathrm{zxy}} E_{\mathrm{x}} E_{\mathrm{y}}^{*} + \chi_{\mathrm{zxy}} E_y E_x^{*} \end{pmatrix} \tag{2}$$

Under a π rotation along *y*-direction (*x*, *y*, *z*→-*x*, *y*, -*z*), the rotation operator *R* can be written as

$$R = \begin{pmatrix} \cos(\pi) & \sin(\pi) & 0 \\ \sin(\pi) & -\cos(\pi) & 0 \\ 0 & 0 & -1 \end{pmatrix} = \begin{pmatrix} -1 & 0 & 0 \\ 0 & 1 & 0 \\ 0 & 0 & -1 \end{pmatrix} \tag{3}$$

In this case, $\overline{J^{\mathrm{LBPVE}}}$ becomes $R\overline{J^{\mathrm{LBPVE}}}$ and (E_x, E_y, E_z) becomes $R(E_x, E_y, E_z)$. Since χ_{ljk} should remain the same under the rotation symmetry, we have

$$R\overline{J^{\mathrm{LBPVE}}}(E_x, E_y, E_z) = \overline{J^{\mathrm{LBPVE}}}(-E_x, E_y, -E_z) \tag{4}$$

Thus, we have

$$\begin{pmatrix} \chi_{\mathrm{xxx}} |E_x|^2 + \chi_{\mathrm{xyy}} |E_y|^2 \\ \chi_{\mathrm{yxy}} E_{\mathrm{x}} E_{\mathrm{y}}^{*} + \chi_{\mathrm{yxy}} E_y E_x^{*} \\ \chi_{\mathrm{zxx}} |E_x|^2 + \chi_{\mathrm{zyy}} |E_y|^2 \end{pmatrix} = 0 \tag{5}$$

Therefore, the BPVE-induced photocurrent along *y*-direction $J_{\mathrm{y}}^{\mathrm{LBPVE}}$ can be expressed as

$$J_{\mathrm{y}}^{\mathrm{LBPVE}} = \chi_{\mathrm{yxx}} |E_x|^2 + \chi_{\mathrm{yyy}} |E_y|^2 \tag{6}$$

Supplementary Note 3

Calculation of IPVE in ReS_2 GBs

Firstly, we constructed the unite cell of ReS_2 with GBs as shown in Supplementary Fig. 14a which contented 56 Re atoms and 112 S atoms. Please note that the unit cell contains both ↑ and ↓ GBs. Since ↑ and ↓ GBs in one unit cell are unequal, the total shift current is nonzero. Secondly, we calculated the band structures, density of states and distribution of electron states using the first-principles calculations method. Then, we fitted the first-principles calculations results by the tight-binding model using the Wannier90-version3.1 software. Because the system needs tremendous computation resources to get a delicate fitting results of such a big system (which costs of weeks), here we only show the best fitted tight-binding results. Please note that there are still some deviations between first-calculation calculations and tight-binding model results which may affect the calculated shift current. Finally, we calculated the shift current in ReS_2 GBs based on the fitted tight-binding model (see Fig. 14b). Along GB direction (*y*-direction), IPVE-induced photocurrents induced by *yxx* tensors dominate the total photocurrent in ReS_2 GBs.

Supplementary References

1. Jiang, J. et al. Flexo-photovoltaic effect in MoS_2. *Nat. Nanotechnol.* **16**, 894-901 (2021).

2. Quereda, J. et al. Symmetry regimes for circular photocurrents in monolayer

$MoSe_2$. *Nat. Commun.* **9**, 3346 (2018).
